# Bragg grating etalon-based optical fiber for ultrasound and optoacoustic detection

**DOI:** 10.1038/s41467-024-51497-1

**Published:** 2024-08-30

**Authors:** Tai Anh La, Okan Ülgen, Rami Shnaiderman, Vasilis Ntziachristos

**Affiliations:** 1 Institute of Biological and Medical Imaging, Helmholtz Zentrum München, Neuherberg, Germany; 2 Chair of Biological Imaging at the Central Institute for Translational Cancer Research (TranslaTUM), School of Medicine and Health, Technical University of Munich, Munich, Germany; 3 Munich Institute of Biomedical Engineering (MIBE), Technical University of Munich, Garching b. München, Germany

**Keywords:** Photoacoustics, Imaging and sensing

## Abstract

Fiber-based interferometers receive significant interest as they lead to miniaturization of optoacoustic and ultrasound detectors without the quadratic loss of sensitivity common to piezoelectric elements. Nevertheless, in contrast to piezoelectric crystals, current fiber-based ultrasound detectors operate with narrow ultrasound bandwidth which limits the application range and spatial resolution achieved in imaging implementations. We port the concept of silicon waveguide etalon detection to optical fibers using a sub-acoustic reflection terminator to a Bragg grating embedded etalon resonator (EER), uniquely implementing direct and forward-looking access to incoming ultrasound waves. Precise fabrication of the terminator is achieved by continuously recording the EER spectrum during polishing and fitting the spectra to a theoretically calculated spectrum for the selected thickness. Characterization of the EER inventive design reveals a small aperture (10.1 µm) and an ultra-wide bandwidth (160 MHz) that outperforms other fiber resonators and enables an active detection area and overall form factor that is smaller by more than an order of magnitude over designs based on piezoelectric transducers. We discuss how the EER paves the way for the most adept fiber-based miniaturized sound detection today, circumventing the limitations of currently available designs.

## Abstract (web summary)

The authors embed a micro-resonator in a 10 µm core fiber, which enables ultrasound wave detection with large bandwidth and small aperture. This facilitates high-resolution optoacoustic (photoacoustic) imaging and ultrasonography.

## Introduction

Offering a unique ability to visualize optical contrast without being significantly affected by photon scattering, optoacoustic (OptA) methods enable high-resolution visualization at depths that go beyond optical microscopy^1^. Proliferating to many different implementations and imaging designs, from basic research to mesoscopic and interventional applications^2–4^, the need for optimized OptA detectors has become greatly apparent. A critical requirement for a high-resolution and image-fidelity OptA mesoscopic implementation is that the detector can collect wide bandwidths, often broader than 100 MHz, to achieve high-resolution imaging beyond the limits of optical or OptA microscopy that uses focused light. In particular, it has been shown that even bands above 120 MHz lead to a better definition of in-vivo optoacoustic mesoscopy images and resolution improvements^5,6^. Therefore, it is imperative that the miniaturization of OptA detectors does not come at the expense of the bandwidth, resolution, sensitivity, and overall performance achieved^7^.

Piezoelectric transducers (PZTs) have been broadly used in OptA applications due to their wide availability. While PZTs could be miniaturized^4,8^, miniaturization reduces the detection sensitivity in a quadratic manner proportional to the area of the sensor^9^, which markedly limits the use of miniaturized PZT elements in OptA applications^10^, as it would require high light intensities that may be damaging for tissue. Miniaturizing detectors based on micromachined fabrication, such as capacitive micromachined transducers (CMUTs), will similarly be limited by the sensitivity achieved. For this reason, the development of optical detection of sound has received particular attention because optical detectors enable a high degree of miniaturization while their sensitivity does not depend proportionally on the sensing area^11–13^. In particular, there is growing interest in optical fiber detectors (OFDs) using interferometry for sound detection^14–16^. OFDs can also allow seamless integration to OptA or multi-modal systems due to their small form factor and the option to be co-localized with an illumination path^16,17^.

Despite the advantages, a significant challenge that limits OFD dissemination in OptA mesoscopy applications is the narrow bandwidth achieved that limits the resolution achieved. Typically combined with large apertures^18^, the resolution that has so far been achieved by OFDs is in the 40 µm range, as summarized in Table 1. Fabry–Perot detectors can reduce the thickness of the external Fabry–Perot cavity to enlarge the detection bandwidth^15^, but at the expense of reduced sensitivity due to a smaller change in optical thickness^19^. In addition, precise fabrication of a thin Fabry–Perot cavity is challenging due to the technical limitations of the polymer deposition process^15^. Fabricating polymer cavities as dense arrays in multicore fibers with Fabry–Perot detector spacings of just above 10 µm^16^ has been also demonstrated. However, this approach rapidly expands the footprint size of Fabry–Perot detectors by more than an order of magnitude while only doubling the spatial resolution, which nevertheless remains insufficient for many OptA mesoscopy applications.

**Table 1:** Comparison of optoacoustic (OptA) detectors for potential miniaturized OptA implementations (see Supplementary Note 1 for details on parameter calculations)

Detector	Configuration	Resolution-related specifications			Sensitivity	Size
		BW [MHz]	Aperture size [µm]	Resolution [lateral × axial, µm]	NEPD [mPa.Hz^−1/2^]	Footprint diameter [mm]
Piezoelectric Detectors	IV PZT^11^	27	Ø 500	500 ×45	450	Ø 1
	High-frequency IV PZT^52^	170	Ø 45	40 ×14	n/a	Ø 1
	Focused PZT^5,53,54^	180	Ø 15	18 ×4	1.8–2.6	>Ø 3
Micromachined Detectors	PMUT array^55,56^	7	100 ×80	1000 ×500	0.5	Ø 1.1
	CMUT array^57^	27	25 ×25	251 ×92	1.8	Ø 1.4
Transparent Detectors	Transparent PZT^58^	42	Ø 130	133 ×29	–	Ø 9
	Polymer microring^59–61^	170	Ø 60	114 ×57	2.1	Ø 2
Silicon Photonics Detectors	Etalon (SWED)^22,23^	230	0.5 ×0.2	16 ×5	9	> Ø 1
	Bragg grating^12^	230	30 ×0.5	16 ×13	9.8	> Ø 1
	Optomechanic ring^13^	36	Ø 20	38 ×33	1.3	> Ø 1
	Chalcogenide ring^62^	220	Ø 40	50 ×44	2.2	Ø 2
Optical Fiber Detectors	Fabry–Perot (single)^15^	28	Ø 30	94 ×66	2.1	Ø 0.125
	Fabry–Perot (array)^16^	50	Ø 30	45 ×31	111–282	Ø 3
	Pi-shifted FBG^14,63^	80	270 ×10	270 ×15	25	Ø 0.125
	EER (sensor herein)	160	Ø 10	14 ×8.5	2.5	Ø 0.125

*BW* Bandwidth, *CMUT* Capacitive Micromachined Ultrasound Transducer, *EER* Embedded Etalon Resonator, *FBG* Fiber Bragg Grating, *IV* Intravascular, *NEPD* Noise Equivalent Pressure Density, *PMUT* Piezoelectric Micromachined Ultrasound Transducer, *PZT* Piezoelectric Transducer, *SWED* Silicon Waveguide Etalon Detector.

A different approach avoids the use of external polymer cavities by using pi-shifted Fiber Bragg Gratings (pFBGs)^14,20^ embedded in optical fibers to introduce internal silica cavities. This class of OptA detector offers high sensitivity due to the high optical Q-factors achieved, despite the resilience of silica to ultrasonic perturbation. pFBGs were characterized to have larger bandwidths and thus finer axial resolutions than those of Fabry–Perot detectors^21^. However, pFBGs only operate in side-looking geometries, wherein the distributive nature of the Bragg reflectors spreads the length of the actual detection aperture over hundreds of micrometers and proportionally smears the lateral resolution. Current high-sensitivity pFBG OFDs offer bandwidths in the range of 50 MHz with effective apertures in the range of hundreds of micrometers, preventing them from reaching microscopy resolutions.

Herein, we investigate the merits of a design that embeds a Bragg grating-based etalon within an Optical Fiber (OF). This design could offer advantageous performance, well beyond the limitations of current state-of-the-art OFDs, by realizing the most wideband detection while offering high sensitivity and the smallest sensing aperture in OF-based OptA detectors to date. The embedded etalon resonator (EER) replaces the millimeter-length Bragg reflectors that were common in previous fiber-based ultrasound detectors with a metallic-based terminator (reflector) that is less than 100 micrometers in length. The thin reflector fundamentally improves the access of the ultrasound wave to the resonating optical cavity, leading to unparalleled coupling efficiency over previous designs. This implementation offers the most direct exposure of the high-intensity optical field in the cavity to ultrasound fields at the OF tip ever achieved. Combined with the confinement of the optical and ultrasound fields (the detection aperture) within the mode field diameter (MFD) of the OF, i.e. a dimension that corresponds to >100 MHz ultrasound frequencies, we postulated that the EER design could offer the highest bandwidth ever achieved by a fiber detector, driving the highest resolution possible by an OptA fiber-based detector. The premise of this development follows recent progress with the world’s smallest ultrasound/OptA detector realized on silicon photonics, which yielded ultra-wide bandwidths (>200 MHz) while measuring less than 0.5 micrometers in size^22,23^, i.e. a detection area close to the diffraction limit of light.

To test the driving hypothesis, we developed an EER within the core of a single-mode fiber. We first investigated the EER’s sensitivity and its dependence on the ultrasonic exposure of the optical field. We then determined the spatial impulse response (SIR) achieved when using the EER as a detector for imaging applications. We theoretically computed the expected SIR using bandwidth and experimentally validated it by measuring the detector’s response to a delta function OptA excitation in time (nanosecond) and in space (2.2 micrometers). Finally, we demonstrate the use of the EER on controlled phantoms and biological samples at laser fluences well below the ANSI limits. We discuss the performance characteristics achieved that are unmatched by any other ultrasound fiber-detector today (see Table 1), in particular, the bandwidth and resolution demonstrated at the smallest aperture from a fiber sensor. We further identify how the proposed design bypasses several challenges of silicon-photonics ultrasound detectors associated with fragile optical connections (packaging)^24^. Then, we discuss future possible implementations that can allow illumination through the EER sensor using a dichroic reflector, an ability not readily available to silicon photonic chips, the latter typically requiring a second optical channel for illumination, which enlarges the overall catheter dimension^7^.

## Results

**Fig. 1:**
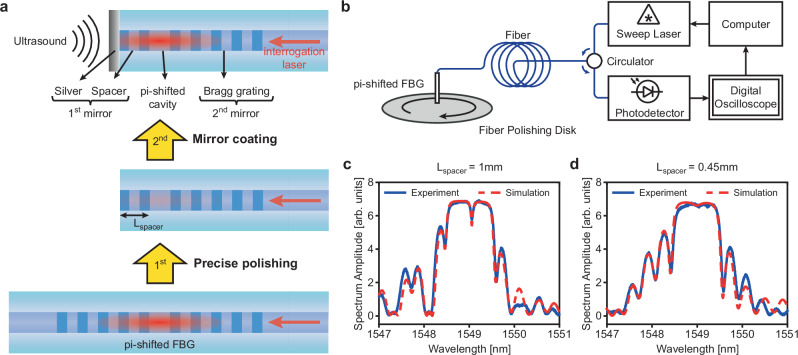
Fabrication process of the Embedded Etalon Resonator (EER) sensor. **a** Structure of the final EER sensor (top), obtained by two fabrication steps: first, a pi-shifted Fiber Bragg Grating (FBG) is precisely polished; second, the polished tip of the fiber is coated with a thin silver mirror. **b** System configuration for precise control of the spacer length in the first fabrication step. Simulated and experimental spectra of the EER with spacer lengths of 1 mm (**c**) and 0.45 mm (**d**) after the first fabrication step.

The EER sensor (Fig. 1a) is based on a Bragg grating-based etalon embedded in the core of a single-mode OF. The etalon comprised of a silver coating terminator and a short FBG section of less than one hundred micrometers in length (spacer), which together served as the first reflective mirror. The second mirror was formed by a 2 mm FBG section. A pi-shifted cavity was sandwiched between the two mirrors to efficiently confine the interrogated optical energy inside the etalon. The etalon was fabricated by precisely polishing away part of a pFBG (1^st^ step) and coating the polished facet with a thin silver mirror (2^nd^ step) (see Methods section “Sensor fabrication” for details). Incident ultrasonic waves interacting with the coated facet induced perturbations and changes in the refractive index of the proximal cavity, which subsequently shifted the resonant frequency of the optical resonator. This shift was probed by recording the intensity of the reflected beam from an interrogating laser at near-resonant radiation. Unlike the Bragg grating used for implementing ultrasound detection on a silicon photonics chip, whereby the effective index of the waveguide was modulated by adding physical corrugation to the waveguide^22^, the Bragg grating within the OF herein was created by a permanent change of the core’s refractive index using UV exposure and has index modulation that is several orders of magnitude weaker than in silicon photonics^25^. As a result, the Bragg grating section of the optical fiber was almost invisible to microscopic imaging, which presented a challenge in controlling the polishing process and raised the risk of over-polishing the pi-shifted cavity. To circumvent this issue, we quantitatively determined the spacer length by spectral fitting of the experimental spectrum obtained from the system depicted in Fig. 1b to a theoretical spectrum obtained by simulation (see Methods section “Spectrum fitting method” for details). Figure 1c, d shows plots of simulated and experimental spectrums for EER sensors after the first fabrication step with spacer lengths of 1 and 0.45 mm, demonstrating how the theoretical simulation helped to guide the fabrication process.

**Fig. 2:**
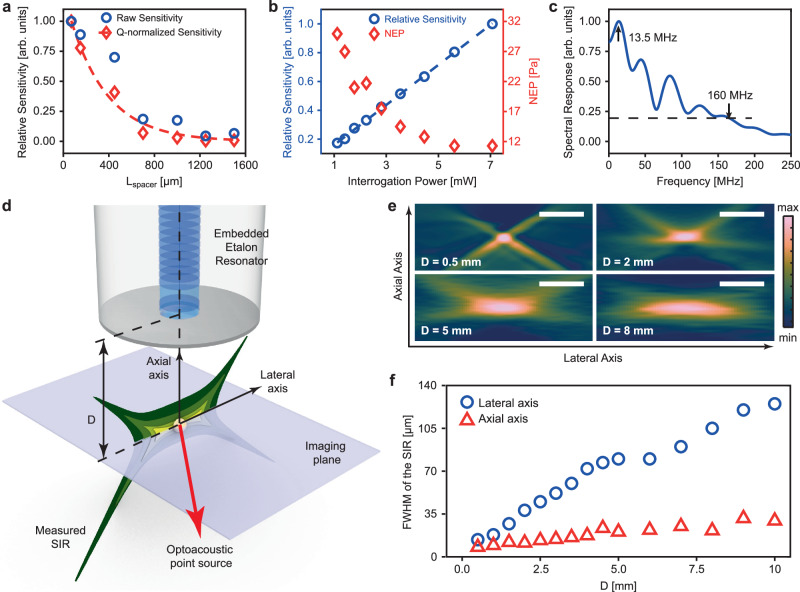
Embedded Etalon Resonator (EER) characterization. **a** The variation of the ultrasonic sensitivity of the EERs with (blue circles) or without (red diamonds) normalization to Q-factor, with respect to the change in spacer length (L_spacer_). The red dashed line represents the simulated optical intensity that is exposed to ultrasound at the sensing facet. **b** Relationship between the sensitivity and noise-equivalent pressure (NEP) with respect to interrogation power. **c** Bandwidth response of the EER. **d** Experiment configuration for spatial response characterization of the EER in optoacoustic mesoscopy. **e** Projection of the measured spatial impulse response (SIR) along the lateral and axial directions at the imaging distances (D) of 0.5, 2, 5, and 8 mm. The scale bar corresponds to 40 µm. **f** Dependence of the full width at half maximum (FWHM) of the SIR on the imaging distance D.

To confirm the hypothesis that the EER detection sensitivity depends on the access of the ultrasound wave to the optical resonator, as it is defined by the distal reflector thickness, we varied the spacer length (See Supplementary Note 2). Guided by the spectrum fitting method, we fabricated several EERs with different spacer lengths and measured their response under the same ultrasound source. The results confirm that the maximum sensitivity is achieved for the smallest spacer length, at 70 micrometers. This maximum sensitivity was observed both in absolute terms and when normalized by the Q-factor of the cavity (Fig. 2a). The Q-factor of each cavity was experimentally determined as described in Supplementary Note 3. The EER sensitivity was also found to be proportional to the intensity of the laser interrogation beam (Fig. 2b) as expected^19^. This finding indicated that at 7 mW of interrogating intensity, the sensor still operated at a linear sensitivity regime. Therefore, it is possible to further increase sensitivity by increasing the intensity of the interrogation beam until reaching non-linearity^26,27^, an ability that could be demonstrated in the future by employing a stronger interrogating light source. By comparison, current demonstrations on optical cavities built on silicon photonics platforms already reached a nonlinear plateau at ∼7 mW interrogating intensities^28^, indicating that the fiber sensor could offer more sensitive detection than Silicon Waveguide Etalon Detector (SWED). As shown in Fig. 2b, we also observed that the noise-equivalent pressure (NEP) of the EER experiences saturation effects with interrogation power above 3 mW due to non-linear effects of the laser noise^29^. This means the NEP of the EER in our system (11.4 Pa or Noise Equivalent Pressure Density (NEPD) of 2.5 mPa Hz^−1/2^ - see Methods section “Sensitivity characterization”) is currently limited not only by the intensity of the interrogating laser but also by the characteristics of the laser noise and can be further enhanced by light sources with better noise behavior or noise reduction methods such as phase read-out^30^, balanced scheme^31^ or pulse interrogation^14^.

The EER detection bandwidth was determined by recording the EER’s response to a broadband OptA point source, generated by focusing a pulsing laser onto a 200-nm-thick gold layer, a method known to enable spatial and temporal responses close to a delta function^32^ (see Methods section “Bandwidth characterization”). The recorded EER signal was transformed to the frequency domain to yield a response of 160 MHz (Fig. 2c), calculated as 20% of the maximum intensity seen in the EER power spectrum; although with a noise threshold at <10% of the maximum, a >200 MHz response can be reported. As expected, the frequency response exhibited a non-uniform pattern, owing to acoustic diffraction at the fiber tip (see Supplementary Note 4). Such ultra-broad detection bandwidth yielded a theoretical axial resolution of better than 8 µm, whereby the lateral resolution was found to be 12.6 µm, due to the ∼10.1 µm size of the EER sensing aperture, i.e. MFD of the chosen waveguide^23^.

The unique performance characteristics found by laboratory characterization of the EER were complemented by examining its SIR. To characterize the spatial characteristics of the EER detection field, we measured its 2D SIR using the experimental arrangement illustrated in Fig. 2d. Herein, the EER was line-scanned over the same OptA point source used for bandwidth characterization. The EER was maintained at a fixed distance from the surface of the gold film (imaging distance D on Fig. 2d - see Methods section “Spatial response characterization”). We observed that the spatial response has elongated diagonal artefacts (Fig. 2e) that are typical diffraction patterns resulting from the convergence and interference between OptA waves^33^, similar to diffraction patterns seen in characterizing the SIR (point spread function) of optical systems. In the axial dimension, the EER spatial response ranged from 8 µm at D = 0.25 mm to 30 µm at D = 10 mm while in the lateral dimension, ranged from 13 µm to 125 µm over the same depth range (Fig. 2f). This lateral spatial sensitivity map is advantageous for point detector configurations since the broadening of the acceptance angle achieved is critical in tomographic imaging for allowing collection of ultrasound waves along projections at different angles within the tissue. The performance seen herein is similar to the performance of the SWED^23^. At D = 0.5 mm, the spatial response was found to be 14 µm in the lateral direction and 8.5 µm in the axial direction, which are in very good agreement with the spatial response predicted from the bandwidth characterization in Fig. 2c.

**Fig. 3:**
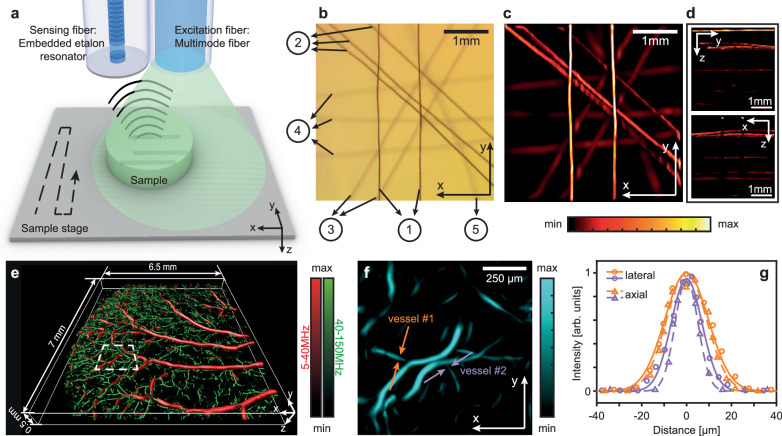
All-optical-fiber optoacoustic (OptA) imaging experiments using the Embedded Etalon Resonator (EER). **a** Experimental imaging setup, wherein the sample is raster-scanned under two optical fibers – one emits diffusive light and the other receives OptA signals. **b** XY-view optical image of the first suture phantom – the numbers in circles indicate different layers of sutures at different depths in the range 0.5–4.5 mm. **c**, **d** Maximum Intensity Projections (MIPs) of the OptA image of the suture phantom in the XY, YZ and XZ planes. **e** Reconstructed image of the vascular structure of a mouse ear. **f** MIP of the rectangular section indicated by a dashed line in **e**. **g** Profiles of the two vessels marked with arrows in **f**. The profiles and the corresponding fits of vessel #1 and #2 are plotted in orange and purple, respectively.

To examine the sensor performance in imaging applications, we employed the EER as the detector for Raster Scan Optoacoustic Mesoscopy (RSOM)^4^ imaging of a controlled phantom. Illumination was provided by a second fiber that ran parallel to the EER (Fig. 3a); the longer dimension of both fibers was only ∼350 µm. The dual fiber configuration was raster-scanned over a first phantom that comprised 12 sutures distributed in five layers (labeled ①-⑤ in Fig. 3b) at depths from 0.5 to 4.5 mm and was embedded in agar gel which mimics the acoustic properties of tissues (see Method section “3D Imaging” for more details of the experiment setup and sample preparation). Images were reconstructed with previously developed 3D inversion in the frequency domain^34^. EER-based images resolved all five suture layers (Fig. 3c, d), demonstrating that the developed sensor can be employed for imaging applications. As expected, the diameter of the sutures appears to increase at different layers as a consequence of the reduction of spatial resolution with respect to imaging depth, due to the steeper attenuation of higher ultrasound frequencies as a function of propagation distance, as shown in Fig. 2f (see also Supplementary Note 6 for quantitative analysis). The sutures’ contrast decreased with increasing imaging depth due to weaker laser fluence and stronger ultrasound attenuation^35^.

To examine whether the EER attained sufficient sensitivity to be employed for biological structures in the place of PZT transducers employed so far in OptA mesoscopy^4^, we also performed imaging of the vascular structure of an ex vivo mouse ear, which is the conventional tissue imaged by OptA systems to demonstrate operation or performance. Capitalizing on its broad bandwidth, EER resolved a broad distribution of micro-vessel diameters ranging from vessels larger than 100 µm, observed in the lower frequencies (e.g. 5–40 MHz - red coded), and smaller vessels (down to <20 µm) at the higher frequencies (e.g. 40–160 MHz - green coded) (Fig. 3e). The blood vessels are visualized due to the light-absorption contrast of hemoglobin, which is abundant in the vasculature. The color coding for the two frequency bands is arbitrary but commonly employed to better visualize smaller vessels in the presence of larger vessels and structures^5,6^. To the best of our knowledge, this is the first time that such a wide range of vessel sizes were resolved with a fiber detector. By zooming in on Fig.3e (area marked with a rectangle), we observed details of the image without separation of the low and high frequency bands (Fig. 3f). By plotting the axial and lateral profiles of two representative vessels (Fig. 3g) we found that vessel #1 had an FWHM of 24 µm in the lateral direction and 22 µm in the axial direction, whereas the smaller vessel #2 was 16 µm in the lateral direction and 12 µm in the axial direction. The difference in the lateral and axial dimensions of these vessels was mostly due to the non-isometric spatial response of the EER and the directional nature of image formation in OptA.

## Discussion

We successfully designed and developed an ultrasound detector, embedded in the core of a single-mode optical fiber, with the highest bandwidth and smallest aperture ever. The developed EER achieved a 3-fold improvement in bandwidth and a 3-fold reduction in the active area compared to any other fiber sensor, leading to resolutions that can be at the order of 10 micrometers. With a total diameter of only 125 µm, the proposed EER offers a significant advance in miniaturization of optical detection of ultrasound using fiber sensors.

Using this design, we also achieved the best OptA spatial resolution ever reported by a fiber sensor. At 14 µm in lateral resolution and 8.5 µm in axial direction, the EER exhibits at least a three-fold resolution improvement compared to state-of-the-art Fabry–Perot detectors^15^ and depending on the implementation even an order of magnitude in improvement compared to pFBG sensors^14^. This considerable enhancement can be attributed to the particular characteristics of the embedded etalon, which features an acoustically homogeneous structure that greatly simplifies the acoustic structure and confines the detection aperture to the MFD. However, compared to chip-based detectors such as SWED or polymer microrings, the EER exhibits a non-ideal frequency response with peaks and dips – which is typical for ultrasound detectors built on fiber tips^36^. It may be necessary to employ methods proposed to reduce this effect of acoustic diffraction by either encapsulating the detector in a rigid support^37^ or smoothening the edge of the fiber tip^38^.

The EER’s design and ease of manufacturing enabled tailorable resolutions. In state-of-the-art Fabry–Perot detectors, clean rooms and high-precision fabrication are necessary to ensure specific cavity thicknesses to achieve the desired bandwidth^15^. In contrast, the EER was fabricated in a conventional optics laboratory environment using only in-house facilities and features spatial resolutions that depend only on the material and dimensions of the OF, which are both strictly controlled by industrial standards. In fact, the fabrication routine of the EER is simple enough that, in the future, it could be automated using fiber polishers and a feed-back circuit (Fig. 1b), making it suitable for large-scale fabrication. Compared to silicon photonics-based OptA detectors, which require highly specialized manufacturing infrastructure, well-trained staff only available at silicon foundries and lead times of several months^39^, the ease of EER manufacturing enables efficient prototyping and fast iterations of OptA detectors. In such a scenario, the major fabrication task, i.e. the creation of a UV mask for pFBG embedding, has a cost that scales down rapidly with the number of sensors produced, potentially lowering the EER’s manufacturing costs to a range comparable to silicon photonics-based OptA detectors.

In addition to good resolution and ease of manufacturing, the use of silica as the cavity material also attains thermal stability, which is an advantageous feature for optical detectors. In general, good thermal stability implies an optical detector with a low thermo-optic coefficient and minimal thermal expansion mismatch between the core and the cladding^40^. Optical fibers are approximately 20-fold less sensitive to temperature than silicon and polymer waveguides^41–43^, which enabled us to continuously operate the EER for several hours in the laboratory environment. The major disadvantage of using silica is the resilience to acoustic perturbation, which herein was compensated by a high Q-factor and strong interrogation power. Unfortunately, both high Q-factor and high-power emission increase the long-term susceptibility of the imaging system to other environmental fluctuations such as mechanical vibration. Therefore, interrogation methods that are immune to environmental fluctuations, such as pulse interferometry^14,44^, are necessary for the operation in real environments, for example intravascular and endoscopic applications.

Similar to other fiber-based sensors, the EER fiber could be also used in the future to guide illumination light for OptA excitation because the metallic mirror currently used can be replaced by a dichroic mirror for transmission of visible and near IR wavelengths^45^. This possibility further miniaturizes the sensor used in OptA sensing or imaging, possibly offering an unparalleled combination of size and resolution. Integration of the illumination into the EER could lead to advantageous overlapping between the illumination and the sensing field, optimally matching illumination and detection from the same volume. The EER is small enough to be integrated into a clinical 3 French-sized endoscope (outer diameter of 1 mm), with space to accommodate other modalities. Therefore, besides offering operation as a point sensor for experimental investigations, it can also be considered in a wide range of applications, for example, as an OptA detector in image-guided laparoscopy or surgery endoscopes possibly using micro electro-mechanical systems (MEMS)-based scanning^46,47^. An alternative method for fabricating 2D EER arrays could employ FBG inscription on multicore optical fibers^48,49^. Fabricating a transparent 2D array of EERs is possible by using the same fabrication routine discussed herein for a single etalon, however, 2D arrays will attain a pitch between detection elements due to the clad thickness of the sensor. Nevertheless, such 2D probes would significantly reduce the amount of physical scanning of either the detector or the imaging object by optically scanning the interrogation beam at the back end of the probes.

In conclusion, by markedly pushing the limits of the positional accuracy of OptA, resolution achieved by fiber-based OptA sensors, and lowering manufacturing costs, the EER may have a profound impact on several fields of miniaturized or point ultrasound and OptA measurements.

## Methods

### Sensor fabrication

The EER was fabricated by slowly polishing one distributed reflector of a commercially available pFBG. The pFBG was purchased from TeraXion Inc., Canada, with a total grating length of 4 mm and a coupling coefficient of κ ≈ 1.4 mm^−1^. The grating was written on a standard polarization-maintaining optical fiber. The pFBG had a band gap of approximately 1 nm and a resonant frequency of around 1549 nm. The pFBG was cut at roughly 5 mm from the position of the grating and polished using standard fiber polishing equipment and diamond lapping film with 3 µm grit to partially remove the grating (1^st^ step). After creating the desired spacer length, the fiber tip was finely polished with a diamond lapping film of 20 nm grit and coated with silver using a wet chemical method involving Ag diamine solution and dextrose, resulting in an Ag thickness of approximately 200 nm^22^ (2^nd^ step).

### Sensor interrogation

To detect the ultrasound signal with the EER, the swept-wavelength tunable laser (ITUNX 1550B, Thorlabs) was locked at an emission wavelength that corresponded to the position of maximum slope in the resonant dip. The reflected signal from the EER was guided to the photodetector (PDB480C-AC, Thorlabs) by an optical circulator (CTR1550PM-APC, Thorlabs). The output signal from the photodetector was digitalized using a high-speed data acquisition card (CSE123G2, Gage).

### Spectrum fitting method

To determine the precise spacer length of the EER during the first fabrication step, the spectrum of the pFBG was continuously recorded during the polishing process through the interrogation of the sweep tunable laser that swept over the stop band of the pFBG, measuring the reflected signal with a fast photodetector and a digital oscilloscope. We simultaneously ran a simulation based on the transfer matrix method^50^, where the length of the second mirror (the unpolished side of the pFBG) was fixed at 2 mm and the length of the polished Bragg reflector (the spacer) was varied. A simple comparison function adjusted the spacer length in the simulation and calculated the difference between the experimental and simulated spectrums. The real spacer length was chosen from the simulations and was the value with the minimum difference between the spectrums. We over-polished several pFBGs to evaluate the uncertainty of the spectrum fitting method and observed that the precision of the spacer length was less than 50 µm at a grating coupling coefficient of κ ≈ 1.4 mm^−1^.

### Sensitivity characterization

To characterize the sensitivity of the EER, its ultrasonic response was calibrated using a 0.5-mm needle hydrophone (Precision Acoustics) in the frequency range of 10–30 MHz. To excite the OptA signals, we used a 515 nm pulsed laser with a pulse repetition rate of 1.2 kHz and pulse width of 1.2 ns (Flare PQ HP GR 2k-500, Innolight) as the illumination source. The ultrasound signal for both the EER measurement and the hydrophone calibration was generated by focusing the output beam of the pulsed laser onto a black vinyl film using a microscope objective (PLN 10x, NA = 0.25, Olympus). The film was glued to a microscope coverslip of 170 µm thickness. The focal spot of the objective was aligned to coincide with the black film and the optical power entering the objective was set to be 310 µW. No signal averaging was made during calibration.

The needle hydrophone was calibrated to have a generally flat spectral response and a sensitivity of approximately 440 mV/MPa in the frequency range of 10–30 MHz. Because a needle hydrophone with sufficient bandwidth for characterization of the EER was not available (i.e., >130 MHz), the NEP of the EER was estimated by the NEP extrapolation method^22^, which was developed to characterize a silicon detector with a smaller aperture and wider bandwidth than the EER. In short, we divided the large bandwidth of our sensor into smaller sections with widths equal to the calibrated band of the hydrophone (20 MHz). Using the fact that we have the same noise level and our point source used for bandwidth characterization emits the same acoustic pressure in all frequencies throughout the detection bandwidth of the EER^32^, we extracted the NEP of the EER at different frequencies from the calibrated band of the needle hydrophone. In the calibrated band of the hydrophone (10–30 MHz), the EER is characterized to have a sensitivity of 145 mV/kPa using 9-dBm interrogation power. The noise level of the EER was monitored as the illumination laser beam path was blocked, giving the root-mean-square noise level of 1.78 mV. The NEP of the EER was then estimated to be 12.4 Pa over the 10–30 MHz range. The peak NEP of the EER was extracted to be 11.4 Pa in the frequency range of 7–27 MHz, which corresponds to the NEPD of 2.5 mPa Hz^−1/2^.

### Bandwidth characterization

To determine the detection bandwidth of the EER, we assessed the sensor response under an ultra-broad-band OptA point source generated by focusing the same pulsed laser used for sensitivity characterization onto a 200-nm-thick gold layer^32^. The lateral optical focus achieved with the objective employed was approximately 2.2 µm, giving an almost ideal point source. The signal recorded from the EER was averaged 1000 times, filtered by a 5–350 MHz digital band-pass filter and converted to the frequency domain using the fast Fourier transform.

### Spatial response characterization

To experimentally validate the spatial response of the EER in OptA mesoscopy, we performed a line scan of the sensor over an OptA point source produced by the same method as previously described in the bandwidth characterization section. The scanning was conducted using a motorized translation stage (MTS50-Z8, Thorlabs). The scanning length was adjusted to fully cover the sensor acceptance angle (100°) as the sensor moved further away from the point source. The step size was set to satisfy the Nyquist-Shannon sampling theorem since the spatial resolutions always comprised of at least five steps. The OptA signal at each step size was averaged 150 times to compensate for the ultra-small signal generated from the point source.

A back-projection algorithm in the frequency domain was applied to reconstruct the signal over the line scan^34^. The spatial responses were determined by measuring the FWHM of the reconstructed SIR.

### 3D imaging

To conduct the imaging experiments, the pulsed laser was coupled into a multi-mode fiber with a core diameter of 200 µm and a cladding diameter of 225 µm (FP200ERT, Thorlabs) for optical illumination. The illumination fiber was placed next to and parallel to the EER, creating an epi-illumination setup. The output beam from the illumination fiber has an average power of 3.2 mW and forms a spot with a diameter of less than 1 mm below the EER. The samples were raster-scanned using a fast 2D scanning stage (MLS203-1, Thorlabs). The scanning step size was 20 µm in both X and Y directions. For the 1 mm × 1 mm section of the mouse ear in Fig. 2f, the step size was set at 2 µm in both X and Y directions. The signals at each step size were averaged 20 times and passed through a 5–200 MHz band-pass filter before reconstruction. To obtain the reconstructed 3D images, 3D inversion in the frequency domain was applied to the time domain signals^34^.

The suture phantoms were made by placing black sutures (Dafilon Polyamide, B. Braun Melsungen) with diameters in the range of 30–39 µm in a mixture of agar gel (2.5% agar powder in water). The phantom was ultrasonically coupled to the detector by deionized (DI) water. 12 sutures were distributed into 5 layers of different depths by carefully placing plastic spacers between suture layers and immobilizing sutures and spacers with tape.

Mice were double-housed and maintained at 22 ± 2^o^C, 55 ± 10% relative humidity and a 12-h light/dark cycle with free access to food and water in the animal facility. An 8 week-old female athymic nude Hsd foxn1 lab mouse (Charles River Laboratories, Sulzfeld, Germany) was randomly selected for the ex-vivo experiments on a mouse ear. All animal procedures were approved by the Government of Upper Bavaria.

The calculated laser energy exposure and laser power exposure in both the suture and mouse ear imaging experiments were around 0.3 mJ/cm^2^ and 0.4 W/cm^2^ respectively, well below (<3%) the safety limit for visible laser radiation according to the American National Standards Institute^51^. No sample damage was observed after the imaging experiments.

### Reporting summary

Further information on research design is available in the Nature Portfolio Reporting Summary linked to this article.

### Supplementary information

Supplementary Information

Peer Review File

Reporting Summary

## Data Availability

The file containing the data of the optoacoustic image is too large to upload to a public repository and can be obtained directly from the authors. The characterization data of the sensor have been deposited in the Zenodo database under the accession code 10.5281/zenodo.12702716.

## Code availability

The algorithms for the spectrum matching method, image reconstruction and data analyses are described in detail in the Methods and in the references. The full image reconstruction and data analysis code are not publicly available because the code is used in licensed technology. The code used for the estimation of the spacer length in the spectrum matching method has been deposited in the Zenodo database under the accession code 10.5281/zenodo.12702681.
