# Supplementary material for: Bragg grating etalon-based optical fiber for ultrasound and optoacoustic detection
Source: Nat Commun. 2024 Aug 30;15:7521. doi: 10.1038/s41467-024-51497-1 (PMC11364814; doi:10.1038/s41467-024-51497-1)
Supplement: Supplementary file 1 — Supplementary Information [file 41467_2024_51497_MOESM1_ESM.pdf]

Supplementary Note 1: Parameter calculations of reported ultrasound detectors

The bandwidths were defined as the upper limit of the 20% of the maximum frequency responses and these values were extracted directly from the literature. Thus, these defined bandwidths are generally larger than the actual bandwidth since most detectors have low-frequency cutoffs above 0 MHz. Nevertheless, the upper limit of a defined detection bandwidth gives a good estimate of the theoretical resolutions[1].

The aperture sizes of the focused Piezoelectric Transducers (PZTs) were approximated to be the same as the focal spot sizes of the transducers, which has the expression[2]:

$$\phi = 1.07 \frac{\upsilon_s}{f_{peak}} F$$

where ϕ is the diameter of the focal spot, υ_s is the speed of sound in the coupling media, f_{peak} is the peak frequency and F is the f-number of the focused transducers.

The apertures of the optical ultrasound sensors were calculated as the area of the projection of the effective mode volume inside the optical resonators onto the detection surfaces of the sensors.

The spatial resolutions if not reported in the literature were calculated using the equations[1]:

$$R_A \approx \frac{0.8\upsilon_s}{f_c}$$

$$\text{and } R_L = \sqrt{{R_A}^2 + {\phi_d}^2}$$

where R_A is the Full Width at Half Maximum (FWHM) of the Spatial Impulse Response (SIR) in the axial direction, f_c is the cutoff frequency of the ultrasound detector, R_L is the FWHM of the SIR in the lateral direction and ϕ_d is the diameter of the detector aperture.

The footprint diameters for all detectors were estimated to be the inner diameters of the smallest possible capsules needed to totally enclose the detectors. For PZTs, the footprints included the associated electronics and housing. For Piezoelectric Micromachined Ultrasound Transducers (PMUTs), Capacitive Micromachined Ultrasound Transducers (CMUTs) and polymer microrings, the footprints were the widths of the substrate of the detectors. For silicon photonics-based detectors, the estimated inner diameters of the capsules contained both the silicon chip and the associated optical packaging. For fiber-based detectors, the footprint diameters were equivalent to the diameters of the optical fibers used to construct the sensors.

Supplementary Note 2: Exposure of the optical field to the ultrasound field

The pi-shifted cavity in the Embedded Etalon Resonator (EER) was sandwiched between two Bragg mirrors, so the optical energy was not tightly confined within the cavity but extended outwards and rapidly decayed toward the two mirrors. Supplementary Figure 1a-c illustrate the simulated optical intensity distributions inside three EERs with spacer lengths of 1.5 mm, 1 mm and 0.45 mm.

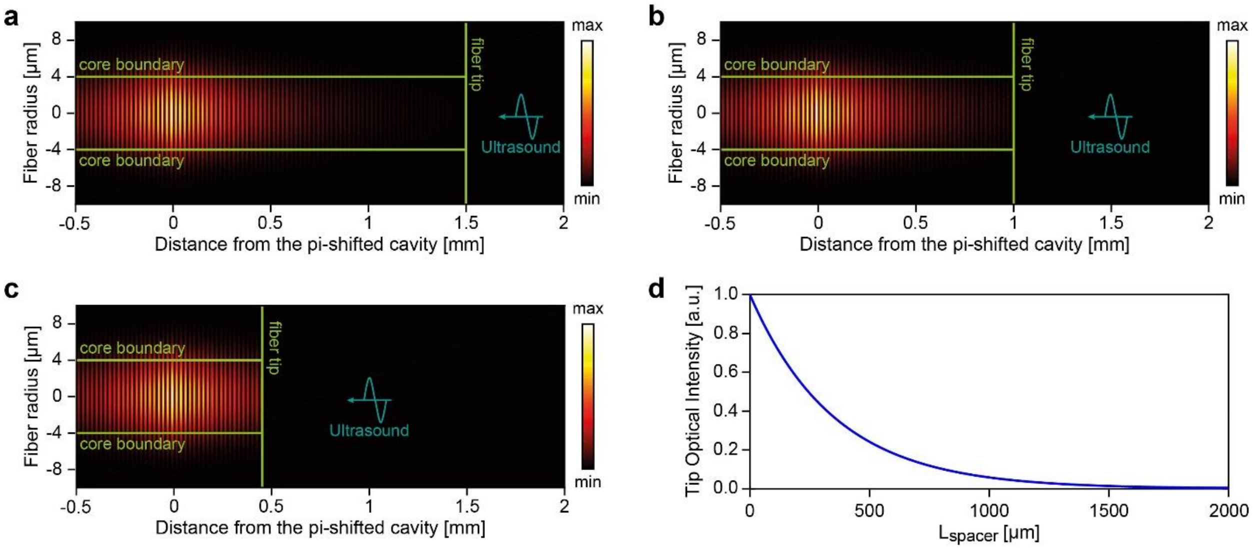

Supplementary Figure 1 – Optical intensity inside the Embedded Etalon Resonator (EER). (a-c) Optical intensity distributions inside the EERs with spacer lengths of 1.5 mm, 1 mm and 0.45 mm. (d) Relationship between the optical intensity at the tip of the EER and the spacer length (L_{spacer}).

As the EER operates in forward-looking directions, ultrasound hits the detector at the tip. Therefore, the optical field exposed to ultrasound should only be in close proximity to the fiber tip. So the three terms: "exposure of the optical field to the ultrasound field", "ultrasonically exposed optical intensity" and "optical intensity at the fiber tip" are used interchangeably within this manuscript. Supplementary Figure 1d depicts the relationship between the theoretical optical intensity at the fiber tip of the EER and the spacer length, assuming the Q-factor remains unchanged. Due to the rapid decaying of the optical intensity outside of the cavity, weaker optical fields were exposed to ultrasound fields when spacer lengths were longer and vice versa. Therefore, for example, by reducing the spacer length of the EER, we could improve the ultrasonically exposed optical intensity.

Theoretically, if the spacer can be totally removed, the optical field measured by the sensor can further be increased by approximately 20% compared to the EER with the shortest spacer length that we fabricated (L_{spacer} = 70 µm). However, due to the uncertainty of our spectrum fitting method, there was a high risk of over-polishing the pi-shifted cavity if the spacer length was shorter than 50 µm. As such, we did not fabricate EERs with spacer lengths shorter than 70 µm.

Supplementary Note 3: Q-factor and Q-normalized sensitivity

To measure the Q-factor of the EERs, the spectrums of the EERs after applying the silver mirror were recorded by a system similar to the spectrum monitoring system depicted in Fig. 1b of the main text. The resonant linewidth was measured from the FWHM of the resonant dip in the spectrum, and optical Q-factors were determined using the approximation:

$$Q = \frac{\delta\nu}{\nu_0}$$

where $\delta\nu$ is the resonant linewidth and ν_0 is the resonant frequency. The Q-factors of the EERs at different spacer lengths are given in Supplementary Table 3.

The first mirror of the EER was comprised of the spacer and the silver coating, so the total reflectivity of the mirror was the combination of the reflectivity of the spacer (R_{spacer}) and the reflectivity of the silver coating. Using the transfer matrix method-based simulation developed for the spectrum fitting process, we calculated R_{spacer} with different spacer lengths and listed the values in Supplementary Table 3. Since the reflectivity of the silver coating remains constant, the reduction of R_{spacer} with respect to spacer length decreased the total reflectivity of the first mirror and subsequently decreased the Q-factor of the EER.

Supplementary Table 1 – Q-factors of the Embedded Etalon Resonators (EERs) with different spacer lengths. Spacer length - L_{spacer}, reflectivity of the spacer - R_{spacer}

L_{spacer} [µm]	1500	1250	1000	700	450	150	70
Q-factor	4.84 x 10^5	4.84 x 10^5	3.69 x 10^5	1.72 x 10^5	1.11 x 10^5	7.38 x 10^4	6.45 x 10^4
R_{spacer} [%]	94.50	89.14	79.13	57.60	31.82	4.40	0.98

The total sensitivity (S) of the EER can generally be expressed as[3,4]:

$$S = \frac{dI}{dP} = \frac{dI}{d\lambda_r}\frac{d\lambda_r}{dn_{eff}}\frac{dn_{eff}}{dP}$$

where I is the optical read-out signal, P is the ultrasonic pressure, λ_r is the resonant frequency and n_{eff} is the effective index of the waveguide. The first term $dI/d\lambda_r$ is the slope of the linear region within the resonant dip and it is linearly proportional to the Q-factor (Q) of the detector. The second term $d\lambda_r/dn_{eff}$ expresses the change of resonant frequency due to changes in the effective index and it can be derived from the standing wave condition of the pi-shifted cavity:

$$\lambda_r = 2L_{cavity}n_{eff}$$

where L_{cavity} is the length of the pi-shifted cavity. So, the second term can be rewritten as:

$$\frac{d\lambda_r}{dn_{eff}} = 2L_{cavity}\,.$$

This term is independent of spacer lengths. The third term dn_{eff}/dP represents the change of the waveguide effective index with respect to pressure. The waveguide effective index changes due to the change in refractive index n of the waveguide materials and the deformation of the physical dimension D of the waveguide. This third term can then be further decomposed into:

$$\frac{dn_{eff}(n,D)}{dP} = \frac{\partial n_{eff}}{\partial n}\frac{\partial n}{\partial P} + \frac{\partial n_{eff}}{\partial D}\frac{\partial D}{\partial P}\,.$$

The two terms $\partial n/\partial P$ and $\partial D/\partial P$ are the pressure-induced changes in refractive indexes and physical dimensions of the waveguide components (i.e., the core and cladding of the Optical Fibers (OFs)), respectively. These two terms depend only on the mechanical properties of the materials used to

construct the OFs (i.e., Young's modulus, photoelastic constant, Poisson's ratio, etc.) and the physical configurations of the waveguide (i.e., dimensions, shape, etc.), but they do not depend on the spacer lengths. The two terms $\partial n_{eff}/\partial n$ and $\partial n_{eff}/\partial D$ take into account the spatial distribution of the confined optical field, meaning that a larger overlap (O) between the optical field and the physical region that is strongly perturbed by ultrasonic pressure results in a better sensitivity[5]. Therefore, the overall sensitivity of the EER can be rewritten as:

$$S(L_{spacer}) = M \times Q(L_{spacer}) \times O(L_{spacer})$$

where M involves all the spacer length-independent parameters. Dividing the raw sensitivity in Fig. 2a of the main text by Q yields the Q-normalized sensitivity, which indicates the dependence of O on the spacer length.

For a probe-type ultrasound detector based on OF, the tip of the OF is the most strongly perturbed region because of the existence of diffractive edge waves and different vibrational modes of the fiber tip in addition to the presence of the longitudinal wave[6]. So, we predicted that the overlap parameter O of the EER greatly depends on the degree of overlap of the optical field with the tip region of the fiber, or the exposure of the optical field to the ultrasound field.

Supplementary Note 4: Frequency response of the Embedded Etalon Resonator (EER)

The frequency response of a fiber optic hydrophone is governed by spatial averaging of the acoustic waves over the dimension of the illuminated volume[7]. These acoustic waves include the incident wave, the reflected waves from acoustic boundaries, the wave transmitted into the fiber, the diffracted wave that originates from the edge of the fiber and propagates in the coupling media adjacent to the detection surface and in the fiber, and the Rayleigh waves produced at the acoustic interface (Supplementary Figure 2). For acoustic waves propagating in the fiber, both shear and longitudinal waves are generated and must be taken into account. These acoustic waves propagate with different speeds of sound and interfere over the optical cavity, resulting in a complex frequency response.

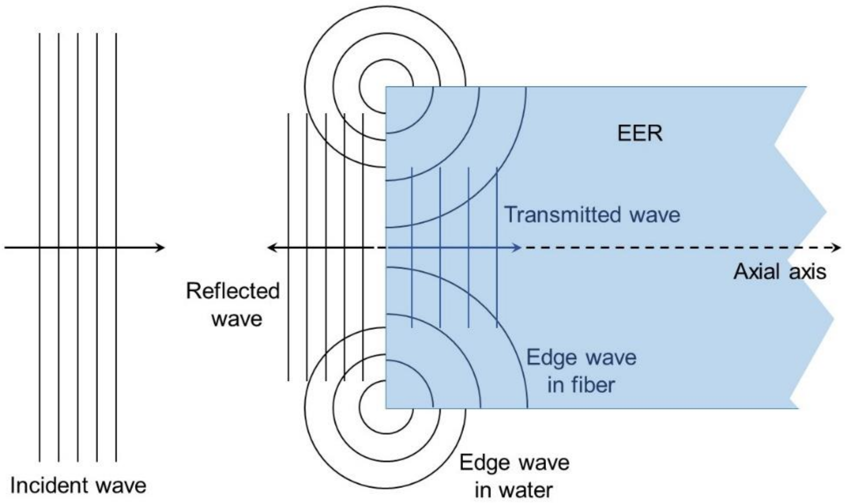

Supplementary Figure 2 – Types of acoustic waves generated from the Embedded Etalon Resonator (EER) under normal incidence.

There are a couple of models developed to study the effect of acoustic interference on the frequency response of optical detectors, but none of those models fit well for the case of the developed Embedded Etalon Resonator (EER). For example, a layered elastic model was developed to explain the frequency response of the polymer Fabry-Perot spacer, but this model did not involve acoustic diffraction and could only be applied to planar detectors [8]. The rigid piston approximation was proposed for the analytical investigation of the Eisenmenger fiber optics hydrophone, and the majority of the sensitivity arises from the penetration of the optical field into the coupling medium[9]. This approximation assumed that the fiber was perfectly rigid and completely neglected the photo-elastic effect and physical deformation of the fiber[10]. For fiber detectors that sensed ultrasound via contraction and extension of the fiber itself, such as the EER, rigorous numerical simulation is required to study the frequency response of the detectors as demonstrated with similar detectors[6,11]. However, some important properties are commonly shared among fiber optics hydrophones:

1. The maximum response is often associated with the resonance of the Rayleigh wave propagating along the fiber tip[7]. This is likely to be the case for the EER because the peak frequency response

(approximately 13.6 MHz) corresponds to the fundamental Rayleigh resonant frequency of the fiber.

2. The upper limit of the detection bandwidth is ultimately limited by the physical dimensions of the acoustically sensitive region, which is determined by the core diameter of the fiber and the thickness of the sensitive region[7]. For the EER, most of the sensitivity arises from the tip region of the fiber, so the effective thickness of the sensing structure is determined as the depth at which surface acoustic waves penetrates beyond the acoustic boundary – which is approximately one acoustic wavelength. Based on the measured bandwidth of the EER, this effective thickness of the EER is estimated to be 20 µm.
3. Increasing the number of acoustic layers greatly complicates the frequency response due to the increment in the number of acoustic waves involved in the interferences. In the case of the EER, the only acoustic boundary is between the coupling medium (water) and silica, so only acoustic propagation in water and silica is considered. However, acoustic propagation in water, silica and polymer must all be considered for Fabry-Perot detectors[8].

Supplementary Note 5: Acoustic attenuation and its effect on the resolution

The acoustic attenuation is frequency dependent, which results in a faster decaying of high-frequency components compared to low-frequency components. This effect led to a reduction in the effective bandwidth of the EER with increasing depth. Here, we used a theoretical approach to account for the effect of acoustic attenuation on the spatial resolution of the EER.

The acoustic attenuation in a wide variety of biomedical media was empirically established to follow the power law[12]:

$$\alpha(f) = \alpha_1 |f|^y$$

where α is the attenuation coefficient [dB/cm], f is the acoustic frequency [MHz]. The two coefficients α_1 and y depend on the media in which the acoustic waves propagate. For water, $y = 2$ and $\alpha_1 = 2.17 \times 10^{-3}$ dB.MHz^{-2}.cm^{-1} while $y = 1.21$ and $\alpha_1 = 0.14$ dB.MHz$^{-1.21}$.cm^{-1} for blood[12]. In most soft tissues, $y = 1$ and $\alpha_1 \approx 0.5$ dB.MHz^{-1}.cm^{-1} [13]. Supplementary Figure 3a presents the change of $\alpha(f)$ in water, blood and soft tissue over the detection bandwidth of the EER.

We integrated acoustic attenuation into the bandwidth response of the EER (Fig. 2c) to obtain the effective bandwidth of the EER as a function of D, which is the distance between the EER and the source. The bandwidths and axial resolutions of the EER as a function of D are plotted in Supplementary Figure 3b and 3c, respectively. It is predicted that the EER possesses a similar resolution in most biological media until D = 2mm. Beyond this distance, the resolution in tissue is expected to be two-fold larger than that in water. The axial resolution appears stair-like due to the non-homogeneous frequency response of the EER detector. Therefore, methods to increase the homogeneity of the frequency response, as stated in the discussion section, are expected to smoothen the relationship between resolution and imaging depth.

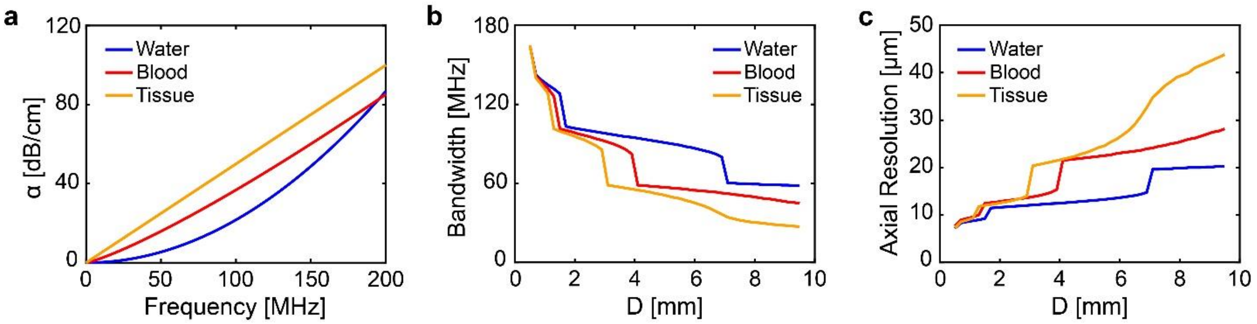

Supplementary Figure 3 – Effect of acoustic attenuation. (a) Acoustic attenuation coefficient (α) in different media. (b, c) Effective bandwidth (b) and axial resolution (c) of the Embedded Etalon Resonator (EER) with respect to imaging distance D.

The reduction of the effective bandwidth of the EER with respect to D also produces an enlargement in the detection aperture due to the increment in size of the effective sensing region with increased acoustic wavelengths[8]. This aperture enlargement is especially obvious in a fiber optic hydrophone – wherein the acoustic diffraction is strong and the sensitive area (the core) is often small compared to the diffracting aperture (the cladding)[10]. Supplementary Figure 4 shows the effective aperture diameter of the EER with respect to imaging distance D, calculated using the measurement of the SIR in Fig. 2f. The effective

aperture enlarges from the size of the mode field diameter to almost the entire diffraction aperture (the outer cladding of the fiber) over a distance range of 1 cm.

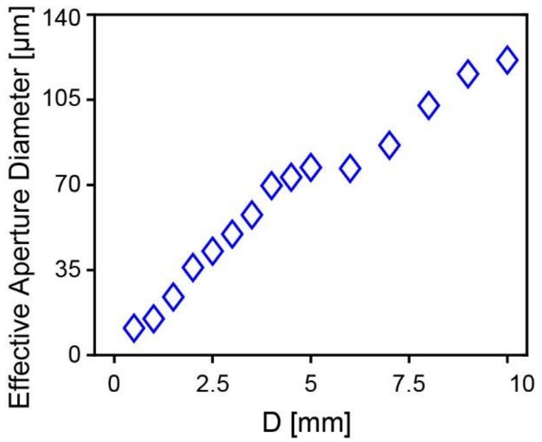

Supplementary Figure 4 – Calculated effective aperture diameter of the Embedded Etalon Resonator (EER) with respect to imaging distance (D).

Supplementary Note 6: Quantitative analysis of the suture phantom experiment

The signal-to-noise ratio (SNR) and spatial dimensions of the sutures at different imaging depths are plotted in Supplementary Figure 5a. We observed a > 10 dB decrement in SNR when the imaging depth was increased from 0.5 mm to 4.5 mm, which can be attributed to the acoustic attenuation and the reduction of laser fluence with respect to imaging depth. Regarding the spatial dimensions of the sutures with respect to the imaging distance D, we witnessed that the smearing rate of the suture's diameters are faster than the deterioration rate of the SIR (Fig. 2f). Several factors contributed to this spatial smearing. First, because the scanning range was fixed at 4 mm, the effective acceptance angle reduced with increasing D and degraded the spatial response[14]. Secondly, the SIR was characterized in water while the sutures were immersed in agar gel, which has similar acoustic properties to tissues. Within the detection bandwidth of the EER (160 MHz), ultrasound attenuates at a faster rate in tissue than in water[13], which decreased the detector's effective bandwidth at increased depths. Thirdly, the illumination fiber had a numerical aperture (NA) smaller than that of the EER (the NA of the illumination fiber was 0.5, and the NA of the EER was characterized to be 0.77). Therefore, part of the effective sensing region of the detector was not illuminated as depicted in Supplementary Figure 5b, resulting in additional smearing of the sutures.

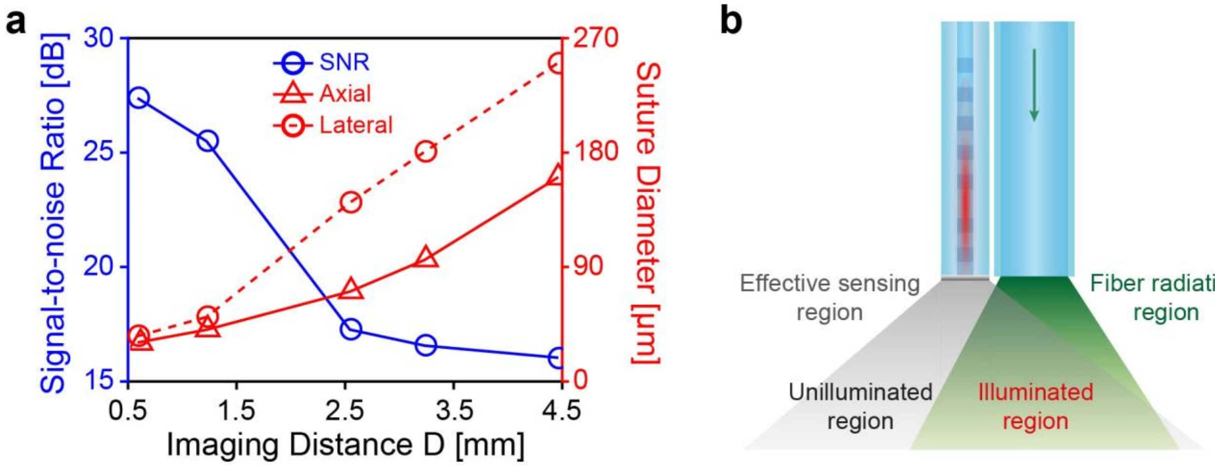

Supplementary Figure 5 – (a) Signal-to-noise ratio (SNR) and diameter of the sutures with respect to imaging depth (D). (b) The radiation region of the illumination fiber compared to the effective sensing region of the Embedded Etalon Resonator (EER).

References

1 Xu, M. & Wang, L. V. Analytic explanation of spatial resolution related to bandwidth and detector aperture size in thermoacoustic or photoacoustic reconstruction. *Physical Review E* **67** (2003). https://doi.org:10.1103/PhysRevE.67.056605

2 Soliman, D. M. *Augmented microscopy: Development and application of high-resolution optoacoustic and multimodal imaging techniques for label-free biological observation* Ph.D. thesis, Technischen Universität München, (2016).

3 Chao, C. y., Ashkenazi, S., Huang, S. w., Donnell, M. O. & Guo, L. J. High-frequency ultrasound sensors using polymer microring resonators. *IEEE Transactions on Ultrasonics, Ferroelectrics, and Frequency Control* **54**, 957-965 (2007). https://doi.org:10.1109/TUFFC.2007.341

4 Li, H., Dong, B., Zhang, Z., Zhang, H. F. & Sun, C. A transparent broadband ultrasonic detector based on an optical micro-ring resonator for photoacoustic microscopy. *Scientific Reports* **4** (2014). https://doi.org:10.1038/srep04496

5 Ravi Kumar, R. *et al.* Enhanced Sensitivity of Silicon-Photonics-Based Ultrasound Detection via BCB Coating. *IEEE Photonics Journal* **11**, 1-11 (2019). https://doi.org:10.1109/jphot.2019.2908013

6 Weise, W., Wilkens, V. & Koch, C. Frequency response of fiber-optic multilayer hydrophones: experimental investigation and finite element simulation. *IEEE Transactions on Ultrasonics, Ferroelectrics, and Frequency Control* **49**, 937-946 (2002). https://doi.org:10.1109/TUFFC.2002.1020164

7 Hurrell, A. B., P. C. in *Ultrasonic transducers: Materials and design for sensors, actuators and medical applications* (ed K. Nakamura) Ch. 19, 619-676 (Woodhead Publishing Limited, 2012).

8 Cox, B. T. & Beard, P. C. The frequency-dependent directivity of a planar fabry-perot polymer film ultrasound sensor. *IEEE Transactions on Ultrasonics, Ferroelectrics, and Frequency Control* **54**, 394-404 (2007). https://doi.org:10.1109/TUFFC.2007.253

9 Staudenraus, J. & Eisenmenger, W. Optisches Sondenhydrophon. *Biomedizinische Technik / Biomedical Engineering* **33**, 105-106 (1988).

10 Krücker, J. F. *et al.* Rigid piston approximation for computing the transfer function and angular response of a fiber-optic hydrophone. *The Journal of the Acoustical Society of America* **107**, 1994-2003 (2000). https://doi.org:10.1121/1.428483

11 Morris, P. *A Fabry-Perot fibre-optic hydrophone for the characterisation of ultrasound fields* PhD thesis, University College London, (2008).

12 Szabo, T. L. *Diagnostic Ultrasound Imaging: Inside Out*. (Elsevier Academic Press, 2004).

13 Deán-Ben, X. L., Razansky, D. & Ntziachristos, V. The effects of acoustic attenuation in optoacoustic signals. *Physics in Medicine and Biology* **56**, 6129-6148 (2011). https://doi.org:10.1088/0031-9155/56/18/021

14 Yuan, X., Dazi, F. & Wang, L. V. Exact frequency-domain reconstruction for thermoacoustic tomography. I. Planar geometry. *IEEE Transactions on Medical Imaging* **21**, 823-828 (2002). https://doi.org:10.1109/TMI.2002.801172
