# Supplementary material for: Bragg grating etalon-based optical fiber for ultrasound and optoacoustic detection
Source: Nat Commun. 2024 Aug 30;15:7521. doi: 10.1038/s41467-024-51497-1 (PMC11364814; doi:10.1038/s41467-024-51497-1)
Supplement: Supplementary file 3 — Reporting Summary [file 41467_2024_51497_MOESM3_ESM.pdf]

nature portfolio

Corresponding author(s): Vasilis Ntziachristos

Last updated by author(s): Jul 16, 2024

Reporting Summary

Nature Portfolio wishes to improve the reproducibility of the work that we publish. This form provides structure for consistency and transparency in reporting. For further information on Nature Portfolio policies, see our Editorial Policies and the Editorial Policy Checklist.

Statistics

For all statistical analyses, confirm that the following items are present in the figure legend, table legend, main text, or Methods section.

n/a	Confirmed	
x		The exact sample size (*n*) for each experimental group/condition, given as a discrete number and unit of measurement
x		A statement on whether measurements were taken from distinct samples or whether the same sample was measured repeatedly
x		The statistical test(s) used AND whether they are one- or two-sided<br>*Only common tests should be described solely by name; describe more complex techniques in the Methods section.*
x		A description of all covariates tested
x		A description of any assumptions or corrections, such as tests of normality and adjustment for multiple comparisons
	x	A full description of the statistical parameters including central tendency (e.g. means) or other basic estimates (e.g. regression coefficient) AND variation (e.g. standard deviation) or associated estimates of uncertainty (e.g. confidence intervals)
x		For null hypothesis testing, the test statistic (e.g. *F*, *t*, *r*) with confidence intervals, effect sizes, degrees of freedom and *P* value noted<br>*Give P values as exact values whenever suitable.*
x		For Bayesian analysis, information on the choice of priors and Markov chain Monte Carlo settings
x		For hierarchical and complex designs, identification of the appropriate level for tests and full reporting of outcomes
x		Estimates of effect sizes (e.g. Cohen's *d*, Pearson's *r*), indicating how they were calculated

Our web collection on statistics for biologists contains articles on many of the points above.

Software and code

Policy information about availability of computer code

Data collection	The code used for the estimation of the spacer length in the spectrum matching method has been deposited in the Zenodo database under the accession code https://doi.org/10.5281/zenodo.12702681.
Data analysis	The algorithms for the spectrum matching method, image reconstruction and data analyses are described in detail in the Methods and in the references. The full image reconstruction and data analysis code are not publicly available because the code is used in licensed technology.

For manuscripts utilizing custom algorithms or software that are central to the research but not yet described in published literature, software must be made available to editors and reviewers. We strongly encourage code deposition in a community repository (e.g. GitHub). See the Nature Portfolio guidelines for submitting code & software for further information.

Data

Policy information about availability of data

All manuscripts must include a data availability statement. This statement should provide the following information, where applicable:

- Accession codes, unique identifiers, or web links for publicly available datasets
- A description of any restrictions on data availability
- For clinical datasets or third party data, please ensure that the statement adheres to our policy

The file containing the data of the optoacoustic image is too large to upload to a public repository and can be obtained directly from the authors. The characterization data of the sensor have been deposited in the Zenodo database under the accession code https://doi.org/10.5281/zenodo.12702716.

Research involving human participants, their data, or biological material

Policy information about studies with human participants or human data. See also policy information about sex, gender (identity/presentation), and sexual orientation and race, ethnicity and racism.

Reporting on sex and gender	n/a
Reporting on race, ethnicity, or other socially relevant groupings	n/a
Population characteristics	n/a
Recruitment	n/a
Ethics oversight	n/a

Note that full information on the approval of the study protocol must also be provided in the manuscript.

Field-specific reporting

Please select the one below that is the best fit for your research. If you are not sure, read the appropriate sections before making your selection.

- [x] Life sciences
- [ ] Behavioural & social sciences
- [ ] Ecological, evolutionary & environmental sciences

For a reference copy of the document with all sections, see nature.com/documents/nr-reporting-summary-flat.pdf

Life sciences study design

All studies must disclose on these points even when the disclosure is negative.

Sample size	A suture phantom was used to validate imaging performance. Using one phantom was deemed sufficient to check performance because the phantom was prepared with sutures at known depths and orientations. One mouse ear was used to test imaging performance. A mouse ear was deemed sufficient because we wanted to test the ability to image randomly-distributed objects and these images could be compared to other images in the literature.
Data exclusions	No data was excluded in this manuscript.
Replication	The vascular network of a mouse ear is unique, so replication was not applicable to this study
Randomization	The mouse and the ear imaged was chosen randomly.
Blinding	A random mouse from the lab was chosen for imaging without any specific requirements, so blinding was not relevant to this study.

Reporting for specific materials, systems and methods

We require information from authors about some types of materials, experimental systems and methods used in many studies. Here, indicate whether each material, system or method listed is relevant to your study. If you are not sure if a list item applies to your research, read the appropriate section before selecting a response.

Materials & experimental systems

n/a	Involved in the study	
x		Antibodies
x		Eukaryotic cell lines
x		Palaeontology and archaeology
	x	Animals and other organisms
x		Clinical data
x		Dual use research of concern
x		Plants

Methods

n/a	Involved in the study	
x		ChIP-seq
x		Flow cytometry
x		MRI-based neuroimaging

Animals and other research organisms

Policy information about studies involving animals; ARRIVE guidelines recommended for reporting animal research, and Sex and Gender in Research

Laboratory animals	An 8 week-old female athymic nude Hsd foxn1 lab mouse (Charles River Laboratories, Sulzfeld, Germany) was randomly selected for the ex-vivo experiments on a mouse ear.
Wild animals	n/a
Reporting on sex	female
Field-collected samples	n/a
Ethics oversight	All animal procedures were approved by the Government of Upper Bavaria.

Note that full information on the approval of the study protocol must also be provided in the manuscript.

Plants

Seed stocks	n/a
Novel plant genotypes	n/a
Authentication	n/a
